# Early reversal of cardiac remodeling in patients with new-onset persistent left bundle branch block after transcatheter aortic valve replacement

**DOI:** 10.1186/s13019-026-03961-w

**Published:** 2026-04-05

**Authors:** Shuzhen Wang, Kunyue Tan, Xiaoqiang Gao, Lijuan Zhang, Chunxia Liu, Xiaofeng Huang, Shuangshuang Yan, Jing Guo, Feng Xiong

**Affiliations:** Department of Cardiology, The Third People’s Hospital of Chengdu, Chengdu, 610031 Sichuan China

**Keywords:** New-onset left bundle branch block, Transcatheter aortic valve replacement, Echocardiography, Cardiac remodeling

## Abstract

**Objective:**

New-onset persistent left bundle branch block (LBBB) following transcatheter aortic valve replacement (TAVR) is an independent predictor of long-term cardiovascular mortality in patients. The aim of this study is to evaluate the impact of new-onset LBBB on early cardiac reverse remodeling and clinical outcomes after TAVR.

**Methods:**

A retrospective analysis was performed on 101 patients who underwent successful TAVR for severe aortic stenosis between March 2021 and October 2024 at our institution. Echocardiographic variables indicative of cardiac remodeling were analysed preoperatively and at one and six months after TAVR. Furthermore, the clinical outcomes of the patients were monitored during the follow-up period.

**Result:**

Of the 101 patients who underwent TAVR, 28 (27.7%) had new-onset LBBB. Transcatheter heart valve(THV) implantation depth was an influential factor for new LBBB, which was more prevalent in patients with thinner interventricular septal thickness preoperatively. At the six-month follow-up, the new-LBBB group showed an increase in left ventricular diameter and left ventricular mass index and a reduction in left ventricular ejection fraction compared with the preoperative period. Mitral regurgitation in the no-LBBB group was significantly reduced at 1 month postoperatively. In contrast, mitral regurgitation in new-LBBB group was reduced at one month postoperatively, but worsened at six months.There was no significant difference in rehospitalization rates within 6 months postoperatively between patients with or without LBBB.

**Conclusions:**

New-onset persistent LBBB after TAVR did not affect short-term rehospitalization, but may adversely affect early postoperative reversal of cardiac remodeling.

## Introduction

Transcatheter aortic valve replacement (TAVR) has become the guideline-directed standard treatment for high-risk surgical patients and an accepted alternative to surgery in lower risk patients with severe aortic stenosis(AS) [1]. Advances in technology, together with accumulated procedural experience, have substantially decreased the incidence of complications, particularly severe paravalvular regurgitation and perioperative mortality. However, cardiac conduction disturbances remain a common complication after TAVR, with left bundle branch block(LBBB) being the most prevalent, with a prevalence of 4%-65% [2]. There are fewer data on the early reversal of cardiac structural and functional remodeling in patients with new-onset LBBB after TAVR. In this study, the effect of new-onset LBBB on early reversal of cardiac structural and functional remodeling in patients after TAVR was analyzed by echocardiography. Additionally, the impact of new-onset LBBB on the short-term rehospitalization of patients was observed.

## Materials and methods

A total of 116 patients with severe AS who underwent successful transfemoral TAVR at our institution between March 2021 and October 2024, all with self-expanding valves, were included. Inclusion criteria were patients with severe AS who had been successfully treated with transfemoral TAVR and were older than 61 years old, with the following diagnostic criteria for severe AS: the effective aortic orifice area <1cm2 or peak aortic valve velocity>4 m/s and mean transvalvular pressure gradient≥40mmHg measured by transthoracic echocardiography [3]. Exclusion criteria comprised patients with a history of LBBB or pacemaker implantation, and those who developed a high degree of perioperative atrioventricular block or had a reversal of LBBB during follow-up. According to these inclusion and exclusion criteria, 3 patients with a history of pacemaker implantation, 7 patients who developed a high degree of perioperative atrioventricular block (5 of whom had pacemakers implanted), and 5 cases of LBBB reversal within 1 month were excluded from this study, and 101 patients were finally included.

New-onset persistent LBBB was defined as LBBB that was not present at baseline but was present on the electrocardiogram(ECG) at discharge(QRS duration > 120 ms, delayed onset of intrinsic deflection in leads V5 and V6, broad monophasic R waves that are usually notched in leads I, V5 and V6, and secondary ST- and T-wave changes in the opposite direction to the main QRS deflection) [4]. The new-LBBB group (28 cases) and the no-LBBB group (73 cases) were categorized according to the presence or absence of new-onset persistent LBBB.

All TAVR procedures were performed under general anesthesia. General clinical and procedural characteristics of the patients were collected. Intraoperative transesophageal echocardiography was used to measure the implantation depth of transcatheter heart valves(THV), which was defined as the distance from the lower edge of the stent to the aortic annulus in the long-axis view [5]. All patients underwent transthoracic echocardiography 1–3 days preoperatively and at 1 month and 6 months postoperatively using an EPIQ 7 C instrument with an S5−1 probe. The structural parameters measured by echocardiography include cardiac chamber diameter, ventricular wall thickness and aortic valve annulus diameter, and calculated of left ventricular mass index. Biplane method was used to measure left ventricular ejection fraction(LVEF). Doppler echocardiography was used to assess hemodynamic parameters, such as peak aortic valve velocity and mean transaortic pressure gradient, to observe valve activity and to grade the degree of mitral regurgitation, which was classified into four grades: no/trace, mild, moderate and severe.Vena contracta (VC) was measured in multiplanar reconstruction mode and the effective regurgitant orifice area (EROA) and regurgitant volume (Rvol) were assessed using transthoracic 2D proximal isovelocity surface area(PISA) method [6]. Worsening or improvement mitral regurgitation was defined as an increase or decrease in the degree of regurgitation by at least one grade or more compared with the previous examination. Paravalvular regurgitation was classified as mild, moderate, or severe according to the ratio of the paravalvular regurgitant bundle to the circumference of the aortic annulus in short-axis views of the aorta [7].

### Statistical analysis

Statistical analysis was performed using SPSS Statistics version 26.0 (SPSS, Inc., Chicago, IL).Continuous variables were tested for normal distribution and chi-squared. Those satisfying both conditions were presented as mean±standard deviation, and intergroup comparisons were performed using independent samples t-tests. Intragroup comparisons before and after surgery were analyzed using F-tests, with multiple comparisons conducted using LSD t-tests. For data that did not meet the assumptions of normality or chi-squared, the median [M(Q25, Q75)] was reported and nonparametric tests were employed: Mann–Whitney U tests were used for intergroup comparisons and Kruskal–Wallis tests for multiple comparisons. Categorical data were presented as frequencies or percentages and intergroup comparisons were analyzed using the Pearson χ^2^ test or Fisher’s exact tests. Multivariate analysis was performed using binary logistic regression analysis. A *P* < 0.05 was considered a statistically significant difference. Data visualization was performed in R (version 4.4.2) with the ggplot2 package to generate stacked bar plots illustrating the temporal trends of mitral regurgitation severity.

## Results

### General clinical characteristics

Among the 101 patients, 28 cases (27.7%) had new-onset persistent left bundle branch block (new-LBBB). The preoperative low-density lipoprotein cholesterol (LDL-C) level was significantly higher in the new-LBBB group than in no-LBBB group (*p* < 0.05). B-type natriuretic peptide (BNP) levels decreased postoperatively in both groups without significant intergroup difference. No significant differences were observed in the remaining baseline clinical characteristics between the two groups (*p* > 0.05). At the 6-month follow-up, there was no statistically significant difference in rehospitalization rates between the groups (*p* > 0.05), as shown in Table 1. No deaths occurred within the 6-month follow-up period.

**Table 1:** Cohort clinical characteristics

Characteristic	No-LBBB(N = 73)	New-LBBB(N = 28)	*P* value
Age(year)	74.20 ± 5.89	71.96 ± 6.02	0.092
Male, *n*(%)	34(46.6%)	14(50.0%)	0.758
NYHA class III-IV, *n*(%)	54(74.0%)	19(67.9%)	0.539
Past History			
Hypertension	59(80.8%)	21(75%)	0.519
Diabetes	17(23.3%)	8(28.6%)	0.582
Atrial Fibrillation	9(12.3%)	4(14.3%)	0.793
Coronary Artery Disease	45(61.6%)	16(57.1%)	0.679
Previous Myocardial Infarction	5(6.8%)	1(3.6%)	0.533
COPD	13(17.8%)	8(28.6%)	0.233
Drug Therapy			
Statin	63(86.3%)	25(89.3%)	0.688
ARNI	9(12.3%)	2(7.1%)	0.454
ACE inhibitors	29(39.7%)	12(42.9%)	0.774
ARB	26(35.6%)	11(39.3%)	0.732
beta-blockers	17(23.3%)	8(28.6%)	0.582
Re-hospitalization within 6 months after surgery	11(15.1)%	6(21.4%)	0.444
Heart Failure	8	4	
Stroke	1	0	
Pacemaker Implantation	0	2	
Pulmonary Infection	2	0	
Laboratory Tests			
Total bilirubin(mol/L)	12.20(9.95, 17.15)	12.31(9.12,15.16)	0.595
Glutamic-pyruvic transaminase(U/L)	17.90(12.20, 26.00)	17.30(15.35,23.52)	0.773
Glutamic-oxalacetic transaminase(U/L)	24.80(21.00, 32.10)	21.60(20.32,30.80)	0.598
Triglyceride(mmol/L)	1.16 ± 0.51	1.31 ± 0.50	0.21
Total Cholesterol(mmol/L)	4.14(3.51, 4.73)	4.91(4.09,5.40)	0.051
High Density Lipoprotein Cholesterol(mmol/L)	1.34 ± 0.30	1.36 ± 0.33	0.778
Low Density Lipoprotein Cholesterol(mmol/L)	2.34(1.90, 2.71)	3.31(2.50,3.77)	0.001
pre-operative BNP(pg/ml)	499.70(91.25, 1286.70)	216.05(35.45,732.77)	0.135
Pre-discharge BNP(pg/ml)	265.00(110.90,604.55)	167.30(98.90,384.67)	0.118
6-month follow up BNP(pg/ml)	156.4(89.55,250.15)	164.85(92.10,233.55)	0.952

Values are represented as Mean (SD) or N (%) or Median [M(Q25,Q75)]

NYHA, New York Heart Association; COPD, Chronic Obstructive Pulmonary Disease; BNP, B-type Natriuretic Peptide; ARNI, Angiotensin Receptor-Neprilysin Inhibitor; ARB, Angiotensin II Receptor Blocker

*P* < 0.05 was considered a statistically significant difference

### Procedural characteristics

The THV implantation depth in the new-LBBB group was significantly lower than that in the no-LBBB group (*p* < 0.05). No significant differences were observed between the two groups in terms of valve type and size, or the procedures involving balloon predilatation and postdilatation, as shown in Table 2.

**Table 2:** Procedural characteristics cetween two groups

Characteristics	No-LBBB(N = 73)	New-LBBB(N = 28)	*P* value
Valve Type			0.944
Venus-A	24(32.9%)	9(32.1%)	
Taurus Elite	49(67.1%)	19(67.9%)	
Valve Size(mm)			0.87
23	12(17.8%)	6(21.4%)	
26	32(43.8%)	13(46.4%)	
29	21(28.8%)	7(25%)	
31/32	8(11%)	2(7.1%)	
Balloon Predilatation	41(56.2%)	20(71.4%)	0.16
Balloon Postdilatation	18(24.7%)	12(42.9%)	0.073
Implantation depth(mm)	3(2,4)	5(4,6)	<0.001

*P* < 0.05 was considered a statistically significant difference

### Comparison of echocardiographic parameters between the two groups

**Graph 1:**
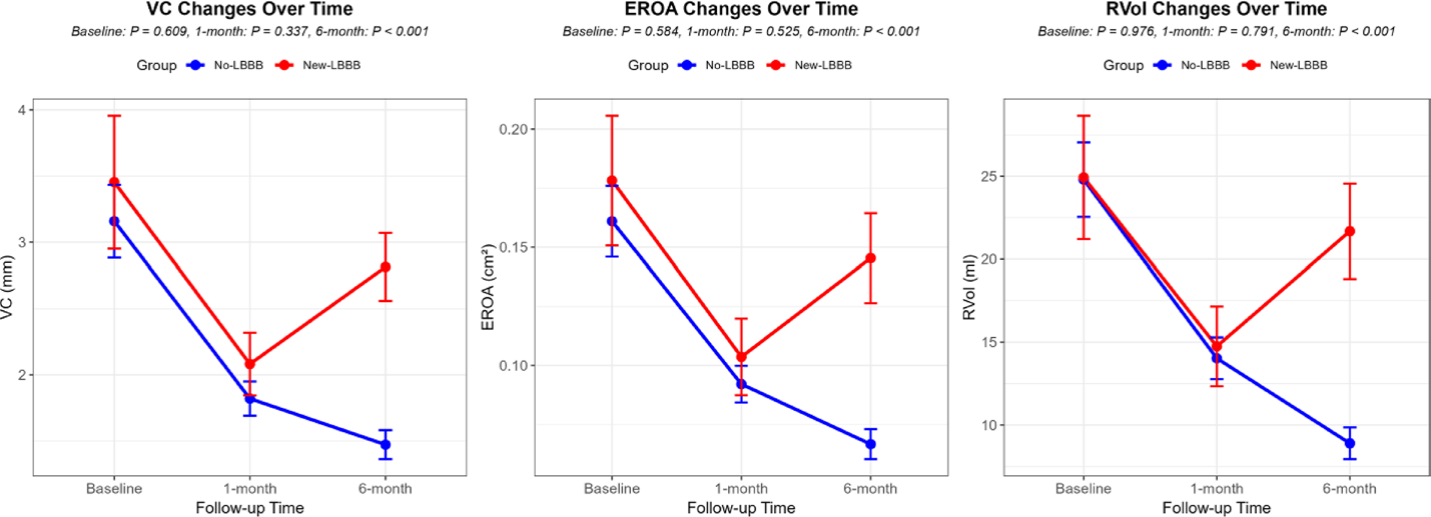
Changes in mitral regurgitation between the two groups after TAVR. There were no significant differences in VC, EROA and Rvol between the two groups at baseline or at the 1-month postoperative follow-up. At the 6-month postoperative follow-up, the VC, EROA and Rvol were found to be larger in the new-LBBB group than in no-LBBB group

Preoperatively, patients with new-onset LBBB showed significantly thinner interventricular septal thickness (IVSd), lower peak aortic valve velocity, and smaller mean transvalvular pressure gradients compared to controls, along with larger effective aortic orifice area (*p* < 0.05). No significant intergroup differences were observed in cardiac chamber diameters, LVEF or diastolic function parameters (*p* > 0.05). Postoperatively, the prevalence of paravalvular regurgitation was comparable between groups (*p* > 0.05). At the 6-month postoperative follow-up, the VC, EROA and Rvol of mitral regurgitation were found to be larger in the new-LBBB group than in the no-LBBB group. However, there were no significant differences in VC, EROA and Rvol between the two groups at baseline or at the 1-month postoperative follow-up, as shown in Table 3 and Graph 1. Binary logistic regression analysis identified THV implantation depth as an independent influencing factor for new-onset LBBB, as shown in Table 4.

**Table 3:** Comparison of Echocardiographic Parameters Between the Two Groups and within each group

Parameters	**New-LBBB Group(N = 28)**			**No-LBBB Group(N = 73)**					
	Baseline	1 Month Follow-up	6 Month Follow-up	Baseline	1 Month Follow-up	6 Month Follow-up	P1 value	P2 value	P3 value
LAD, mm	41.10 ± 5.82	41.85 ± 5.03	42.71 ± 4.95	41.93 ± 5.17	40.89 ± 4.83	39.06 ± 3.66▲	0.491	0.526	0.001
LVEDD, mm	46(42.5,53.75)	47.50(45,50.75)	51.5(49,55.5)▲	48(43, 54.5)	46(44,52)	44(42,48)▲	0.643	0.001	0.001
LVESD, mm	32(28.25,37)	32(29,36)	35(31.5,40)	33(28.5, 39)	31(29, 36)	31(29,33)▲	0.684	0.131	0.025
IVSd, mm	12(12,14)	13(12,13.75)	13(12,15)	13(12,14.5)	13(12,14)	12(12,14)▲	0.041	0.351	0.022
LVPWd, mm	12(11,13)	12(12,13)	13(12,13)	12(12,14)	12(11,13)	12(11,13)	0.239	0.051	0.224
LVMI(g/m^2^)	146(115.75, 188)	148(127, 183.75)	190(144.25, 213.25)▲	160(129,197.5)	145(126, 179)	136(116, 152)▲	0.260	0.018	<0.001
LVEF,%	60(53, 61.75)	57.5(55, 60.75)	54(52.25, 57)▲	58(53, 62)	59(56.5, 62)	61(59,63)▲	0.828	0.034	0.008
RVD, mm	21.32 ± 1.96	21.21 ± 2.07	21.28 ± 2.03	20.89 ± 2.03	20.82 ± 2.20	21.28 ± 2.73	0.008	0.98	0.433
RAD, mm	37.64 ± 5.83	37.78 ± 4.58	38.17 ± 5.07	37.41 ± 5.93	37.45 ± 6.27	36.67 ± 4.37	0.86	0.923	0.639
Aortic annulus diameter, mm	22(20,23)			22(21,23)			0.144		
Peak aortic valve velocity, m/s	4.51 ± 0.47	2.22 ± 0.43▲	2.20 ± 0.46▲	4.71(4.23,5.54)	2.20(1.98,2.51)▲	2.25(2.00,2.41)▲	0.023	<0.001	<0.001
Mean PG, mmHg	47.00 ± 11.17	9.42 ± 2.61▲	9.85 ± 2.63▲	53(41.5, 70.5)	10(8, 11)▲	10(9,11)▲	0.035	<0.001	<0.001
Effective aortic orifice area, cm2	0.86(0.74,0.93)			0.73(0.57,0.90)			0.046		
Mitral valve E-wave velocity, cm/s	70(61.5,103.50)	89(78.75,101.25)	83.5(66.25,106.75)	74(58,96)	85(65.5,108.5)	85(68.5,103.5)	0.961	0.098	0.08
Early diastolic mitral annular velocity e’,cm/s	5(4, 6)	5(4, 6.75)	5(4,6.75)	5(4, 6)	5(4, 6)	6(4, 7)▲	0.516	0.295	0.001
E/e’	15(11.50,19.75)	19.5(13,22.75)	15.5(12.25,19)	18(12,21)	17(14,23)	15(12,19)	0.356	0.3	0.055
Cases with LVEF < 50% (n, %)	5(17.9%)	4(14.3%)	6(21.4%)	16(21.9%)	10(13.7%)	4(5.5%)▲	0.653	0.784	0.015
Mitral Regurgitation							0.933	0.002	<0.001
No/Trace	5(17.9%)	18(64.3%)▲	11(39.3%)	13(17.8%)	44(60.3%)▲	46(63%)▲			
Mild	16(57.1%)	8(28.6%)▲	7(25.0%)▲	40(54.8%)	26(35.6%)▲	25(34.2%)▲			
Moderate	4(14.3%)	2(7.1%)	9(32.1%)✱	14(19.2%)	3(4.1%)▲	2(2.8%)▲			
Severe	3(10.7%)	0(0%)	1(3.6%)	6(8.2%)	0(0%)▲	0(0%)▲			
Vena contracta, mm	2.5(2.07,5.10)	1.95(1.35,2.5)▲	2.4(2.0,3.5)	2.3(2.05,4.25)	2.0(1.0,2.35)▲	1.3(1.0,2.0)▲	0.609	0.013	<0.001
Efficient regurgitant orifice area, cm2	0.13(0.10,0.25)	0.08(0.05,0.14)▲	0.10(0.08,0.23)	0.12(0.10,0.21)	0.08(0.04,0.13)▲	0.05(0.03,0.10)▲	0.584	0.049	<0.001
Regurgitant volume, ml	19(15.25,36.25)	10.50(7.25,23.25)▲	16(10.25,35))	20(15,32)	11(7,21)▲	7(2,15)▲✱	0.976	0.041	<0.001
Paravalvular regurgitation		3(10.7%)			10(13.7%)		0.688		
Mild		3			9				
Moderate		0			1				
Severe		0			0				

Values are represented as Mean (SD) or N (%) or Median [M(Q25,Q75)]

(▲) represents a statistically significant difference compared to preoperative within the same group; (✱) indicate a statistically significant difference within the group between the 6-month and 1-month postoperative follow-ups

LAD, left atrial diameter; LVEDD, left ventricular end-diastolic diameter; LVESD, left ventricular end-systolic diameter; IVSd, Interventricular Septum thickness at end-Diastole; LVPWd, Left ventricular posterior wall thickness; LVMI, left ventricular mass index; LVEF, left ventricular ejection fraction; RVD, right ventricular diameter; RAD, right atrial diameter; Mean PG, mean transaortic pressure gradient

P1 represents the comparison of preoperative parameters between the New-LBBB group and No-LBBB group

p2 represents the comparison of pre- and post-operative parameters within the New-LBBB group

P3 represents the comparison of pre- and post-operative parameters within the No-LBBB group

**Table 4:** Binary Logistic Regression Analysis

Parameters	β	S.E	wald	OR	95%CL	*P* value
Prosthetic valve implantation depth	1.079	0.248	18.878	2.941	1.808–4.785	<0.001
Interventricular septal thickness	-0.109	0.223	0.237	0.897	0.580–1.389	0.626
Mean transaortic pressure gradient	-0.028	0.03	0.871	0.972	0.917–1.031	0.351
Effective aortic orifice area	0.064	2.502	0.001	1.066	0.008-143.846	0.98

### Intra-group comparison of echocardiographic parameters before and after surgery

**Fig. 1:**
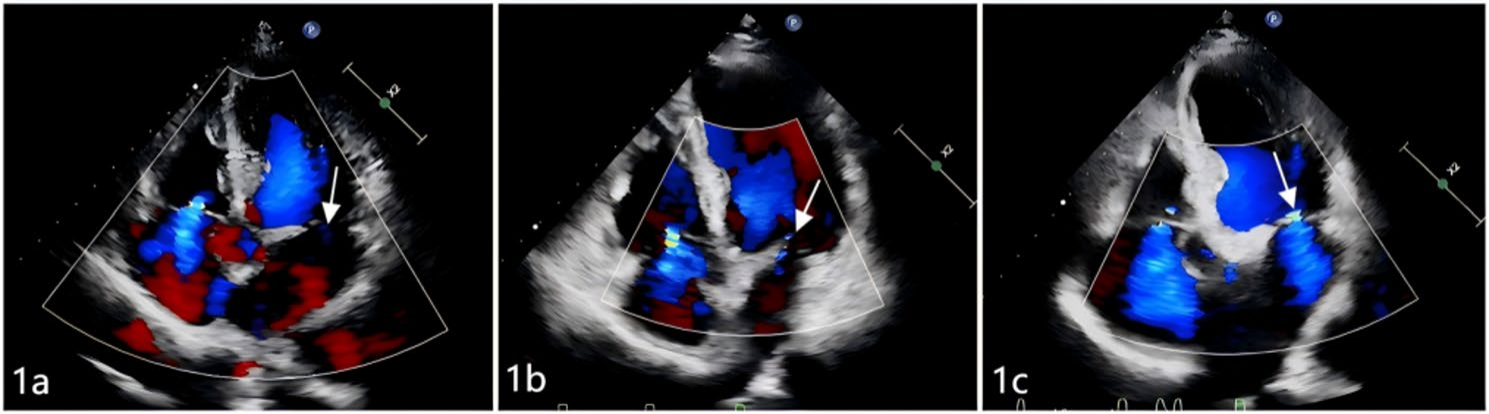
Echocardiography of a patient in the New-LBBB group: (**a**) shows trace mitral regurgitation before the operation; (**b**) shows trace mitral regurgitation 1 month after the operation; (**c**) shows mild mitral regurgitation 6 months after the operation, as the arrow points

**Fig. 2:**
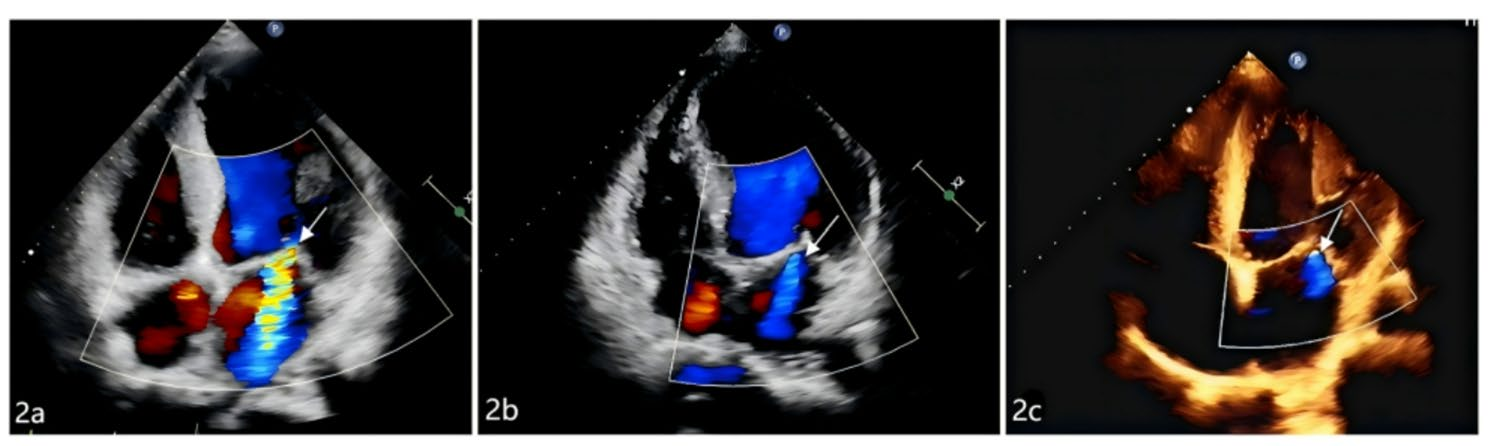
Echocardiography of a patient in the no-LBBB group: (**a**) shows severe mitral regurgitation before the operation; (**b**) shows mild mitral regurgitation 1 month after the operation; (**c**) shows trace mitral regurgitation 6 months after the operation, as the arrow points

**Graph 2:**
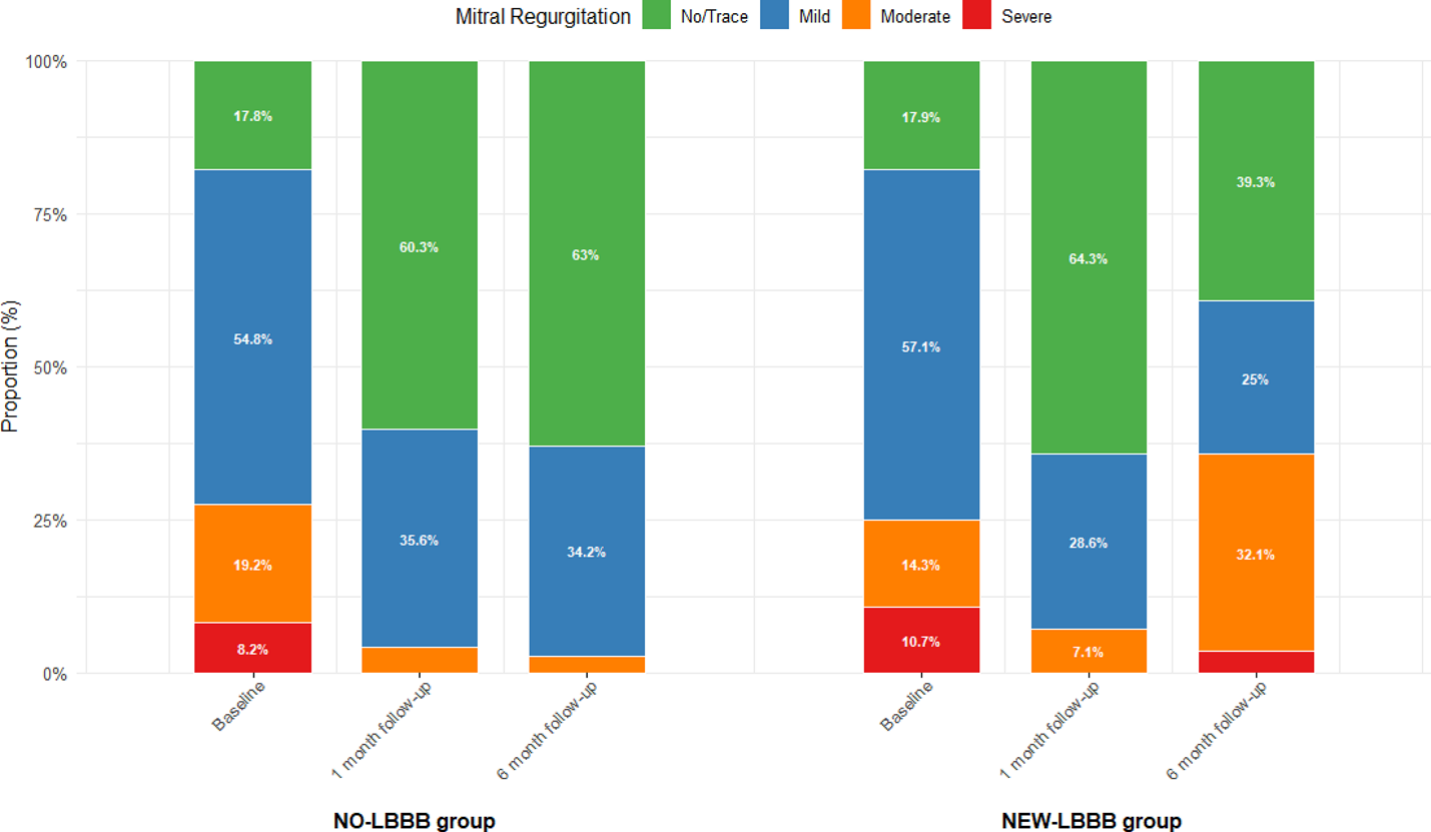
Proportion of Mitral Regurgitation Severity Across Different Time Periods.At 1-month follow-up, the proportion of moderate and severe mitral regurgitation decreased significantly in both groups compared with preoperative levels. At 6-month follow-up, the proportion of moderate regurgitation increased in the NEW-LBBB group compared with the 1-month follow-up, while the degree of regurgitation remained stable in the NO-LBBB group

At the 1-month postoperative follow-up, both groups exhibited significant reductions in peak aortic valve velocity and mean transvalvular pressure gradient compared with preoperative values(*P* < 0.05), while no significant differences were observed in cardiac chamber diameters, left ventricular mass index (LVMI), LVEF, or diastolic function parameters (*P* > 0.05). By the 6-month follow-up, the new-LBBB group demonstrated significant increases in left ventricular end-diastolic diameter and LVMI, accompanied by a reduction in LVEF compared to preoperative levels (all *p* < 0.05). In contrast, the no-LBBB group showed favourable reverse remodeling, characterized by significant decreases in left atrial and left ventricular diameters, reduced interventricular septal thickness, lowered LVMI, and improved LVEF, along with a smaller proportion of patients presenting with LVEF < 50% and an increased early diastolic mitral annular velocity (e′) (all *p* < 0.05), as shown in Table 3. Regarding mitral regurgitation, both groups showed significant improvement at the 1-month follow-up, with a reduced proportion of moderate-to-severe cases and decreases in VC, EROA, and Rvol compared to preoperative levels (all *p* < 0.05). At the 6-month follow-up, the new-LBBB group exhibited an increase in the proportion of moderate regurgitation. Although VC, EROA, and Rvol in this group remained below preoperative values, they demonstrated a rising trend relative to 1-month measurements (all *p* > 0.05). In contrast, the no-LBBB group maintained overall stable regurgitation severity, with Rvol continuing to decline compared to the 1-month level, while reductions in VC and EROA between these two time points were not statistically significant, as shown in Table 3, Graph 2, Figs. 1 and 2.

## Discussion

The results of this study are as follows: ① The incidence of new-onset LBBB after TAVR is about 27.7%; ② Lower THV implantation depth is an independent influencing factor for new-onset LBBB; ③ Although new-onset LBBB does not significantly impact short-term rehospitalization rates, it is an adverse factor for the early reversal of cardiac structural and functional remodeling after TAVR.

New-onset LBBB is the most common conduction disturbance following TAVR, with an incidence ranging from 18% to 64% for self-expanding valves [2]. The development of new-onset LBBB is associated with impaired outcomes after TAVR, mainly including prolonged hospitalization, an increased risk of pacemaker implantation, a higher rehospitalization rate, and poorer survival, which puts a burden on the healthcare system that cannot be ignored [8, 9]. However, this study found that the incidence of new-LBBB was approximately 27.7%, consistent with previous studies using self-expanding valves [2]. This study found no adverse effect of LBBB on patient prognosis, which may be related to the short follow-up period.

TAVR-associated conduction disturbances have been demonstrated to be predominantly caused by direct mechanical injury to the conduction system by the THV device, and to a lesser extent, may be related to local septal hematoma or myocardial ischemia. Pre-existing left anterior hemiblock and membranous septum length are the main influencing factors [9, 10]. Our study identified a lower implantation depth of the THV as an independent predictor of new-onset LBBB following TAVR. Lower implantation depth increases mechanical compression on the basal interventricular septum, increasing conduction disturbance risk [11].

AS leads to LV pressure overload, LV hypertrophy, and impaired myocardial contractility, making reverse remodeling a key therapeutic goal in these patients. Patients with reduced LVEF demonstrate immediate postprocedural LVEF improvement [12]. This study showed that the no-LBBB group exhibited reverse remodeling, characterized by a reduction in cardiac chamber diameter, a decrease in LVMI, and an increase in LVEF at six months postoperatively. Studies have demonstrated that reverse remodeling, characterized by reductions in LVMI and relative wall thickness, can occur within 30 days after TAVR. Furthermore, a greater degree of LVMI reduction is associated with a lower 1-year hospitalization rate, and each 10% reduction in LVMI is associated with a 5% decrease in all-cause mortality over 2–5 years of follow-up [13]. In this study, patients with new-onset LBBB exhibited increased LVMI, decreased LVEF and worsened mitral regurgitation at 6 months postoperatively, suggesting that new-LBBB is an adverse factor for early reverse cardiac remodeling after TAVR.This is consistent with the findings of Kim et al. [14], who observed increased LV diameter and decreased LVEF at 1 year after TAVR in patients with new-onset LBBB. Mechanistically, LBBB alters the sequence of ventricular depolarization and repolarization, leading to uncoordinated ventricular contractions and LV contractile inefficiency. The septal contribution to LV systolic function is attenuated, placing an excessive workload on the LV free wall, which responds with maladaptive remodeling or even decompensation [15]. In the present study, we observed a significant reduction in mitral regurgitation in both groups within one month postoperatively. This is consistent with previous findings where Abdelghani M et al. found that 60% of patients with moderate to severe mitral regurgitation had a significant reduction at 1 month after TAVR [16]. This initial benefit is primarily attributed to the immediate relief of left ventricular pressure overload and the consequent decrease in the left ventricle-to-left atrium pressure gradient following TAVR [17]. However, our serial echocardiographic follow-up at six months showed a different result. Patients with new-onset LBBB exhibited a worsening trend in mitral regurgitation severity, while those without LBBB showed a continued reduction in regurgitation. The reduction in LV diastolic volume, leading to a decrease in mitral tethering, is the main factor for the further reduction in regurgitation in the later period [18].This deterioration may be driven by LBBB-specific pathophysiology. Asynchronous ventricular activation can lead to adverse remodeling, ventricular dilatation, and dyssynchronous papillary muscle contraction, thereby exacerbating mitral valve tethering [15, 19].

The present study showed that the interventricular septum was thinner in the new-LBBB group, a finding for which we propose the mechanism of inadequate cushioning against THV extrusion. This stands in contrast to the more prevalent focus on septal thickening. Prior studies have indicated that a significantly thickened septum can narrow the left ventricular outflow tract, increasing procedural complexity and potentially exacerbating mechanical injury to the conduction system, thereby leading to conduction disturbances [20]. However, Kiefer et al. found that basal septal thickening increased only the need for postoperative balloon dilatations without raising the 30-day incidence of LBBB [21]. Conversely, Pravda et al. suggested that increased basal septal thickness might act as a protective anatomical barrier for the conduction system during TAVR [22]. These divergent findings are likely attributable to the well-documented anatomical variations in the spatial relationship between the conduction system and the muscular ventricular septum [23]. We found that patients in the new-LBBB group had higher levels of low-density lipoprotein cholesterol (LDL-C). Elevated low-density lipoprotein cholesterol (LDL-C) promotes fibrocalcific remodeling and exacerbates coronary ischemia, which may consequently increase the risk of conduction system impairment [24].We also observed a rapid postoperative decrease in BNP levels in both groups. This is likely attributable to the elevated cardiac stress induced by severe AS, which stimulates BNP synthesis. Following TAVR, the reduction in atrial and ventricular pressures leads to a decrease in the stress, consequently resulting in lower BNP production [25].

It should be noted that this study has several limitations. First, this was a single-center surgical experience, which may introduce selection bias. Secondly, the technology of our centre is in the stage of accumulating experience, the skill of the operating physicians may have a certain impact on the incidence of new LBBB. Thirdly, the study was a short-term follow-up, and the results of the indicators cannot reflect long-term outcomes, which require further observation.

In conclusion, while new-onset LBBB following TAVR was not associated with worse short-term clinical outcomes in this cohort, it appears to be an adverse factor for early reverse cardiac remodeling, potentially portending poorer long-term structural and functional recovery.

## Data Availability

No datasets were generated or analysed during the current study.
